# Foliar fungi-imposed costs to plant productivity moderate shifts in composition of the rhizosphere microbiome

**DOI:** 10.3389/fpls.2025.1558191

**Published:** 2025-03-05

**Authors:** Brett R. Lane, Molly A. Kuhs, Max M. Zaret, Zewei Song, Elizabeth T. Borer, Eric W. Seabloom, Daniel C. Schlatter, Linda L. Kinkel

**Affiliations:** ^1^ Department of Plant Pathology, University of Minnesota, St. Paul, MN, United States; ^2^ Department of Ecology, Evolution and Behavior, University of Minnesota, St Paul, MN, United States; ^3^ Plant Science Research Unit, United States Department of Agriculture – Agricultural Research Service (USDA-ARS), St. Paul, MN, United States

**Keywords:** soil ecology, plant-microbe interactions, rhizodeposition, productivity, rhizosphere microbiome

## Abstract

Plants in grasslands navigate a complex landscape of interactions including competition for resources and defense against pathogens. Foliar fungi can suppress plant growth directly through pathogenic interactions, or indirectly via host growth-defense tradeoffs. The exclusion of foliar fungi allows the reallocation of resources from defense to growth and reproduction. In addition, plants also invest photosynthates in rhizodeposition, or root exudates, which play a significant role in shaping the rhizosphere microbial community. However, it remains unclear what impact the exclusion of foliar fungi has on the allocation of resources to rhizodeposition and the composition of the rhizosphere microbial community. Using a 6-year foliar fungicide study in plots planted with 16 species of native prairie plants, we asked whether foliar fungi influence the rhizosphere microbial composition of a common prairie grass (*Andropogon gerardii*) and a common legume (*Lespedeza capatita*). We found that foliar fungicide increased aboveground biomass and season-long plant production, but did not alter root biomass, seed production, or rhizosphere microbial diversity. The magnitude of change in aboveground season-long plant production was significantly associated with the magnitude of change in the rhizosphere microbial community in paired foliar fungicide-treated *vs*. control plots. These results suggest important coupling between foliar fungal infection and plant investment in rhizodeposition to modify the local soil microbial community.

## Introduction

Perennial grasslands sequester significant quantities of soil carbon and play a major role in global nutrient cycling (Bai and Cotrufo, 2022). In contrast to forest ecosystems, grasslands sequester the bulk of their stored carbon (up to 98%) belowground, a form that is resistant to major disturbances, including drought, heat stress, and wildfires (Allen et al., 2010; Anderegg et al., 2015; Dass et al., 2018; Hungate et al., 1997; McDowell and Allen, 2015; Vicente-Serrano et al., 2013). Grassland productivity, the primary source of stored carbon, can be underestimated if it does not account for losses to plant-heterotroph interactions, including the effects of plant pathogens and other herbivores (Duffy et al., 2007; Seabloom et al., 2017). Foliar fungi, including pathogenic and non-pathogenic species, may limit plant biomass accumulation via direct effects such as the consumption of plant photosynthates and biomass, as well as indirect effects including altered plant resource allocation reflecting potential growth-defense tradeoffs.

Pathogenic fungi can reduce photosynthesis by producing lesions and chlorotic areas on infected leaves (Carretero et al., 2011; Kumudini et al., 2008). Plant responses to pathogen infection, such as lignin deposition and the production of reactive oxygen species, also can necessitate the reallocation of resources from growth and competition to defense (Cesarino, 2019; Huot et al., 2014; O’Brien et al., 2012). While non-pathogenic fungi (e.g., endophytes) can provide protection from abiotic stresses including drought, heat, and salt (Rodriguez et al., 2009), they also capture nutrients from the host plant potentially imparting a fitness cost upon the host plant. In particular, some non-pathogenic foliar fungi can prime the plant immune system for a more rapid and stronger response to pathogen infection (Pieterse et al., 2014). While the fitness cost of defense priming on the host plant is lower than constitutively-activated defenses, the costs can impose a growth-defense tradeoff (Van Hulten et al., 2006; Walters et al., 2008).

Fungicide applications reduce fungal infections, alter the aboveground host microbiome (Lane et al., 2023b; Ruuskanen et al., 2023; Yu et al., 2023), and can increase aboveground host biomass, in part by allowing plants to reallocate resources previously used for defense into host growth and productivity and by reducing carbon losses to foliar microbial populations (Seabloom et al., 2017). Traditional frameworks of plant biomass allocation posit that these resources could be reallocated among leaves, stems, roots, and reproduction (Bazzaz and Grace, 1997). However, plants also may invest a significant proportion of their photosynthates in rhizodeposition, or the release of carbon compounds into the surrounding soil from living plant roots (Jones et al., 2004), with potential impacts on the composition of the rhizosphere microbial community (Tian et al., 2020; Vives-Peris et al., 2020).

The rhizosphere, the soil directly adjacent to plant roots, is shaped by interactions between roots and microorganisms. Rhizodeposition may benefit the host plant by facilitating soil microbial nutrient cycling, by stimulating microbial populations that promote host growth, or by suppressing soilborne pathogens (Bais et al., 2006). However, rhizodeposition may alternatively harm the host directly by favoring pathogenic soil microbes, indirectly by reducing disease-suppressing soil microbes, or by promoting the growth of microbes which compete with plant roots for nutrients (Dotaniya and Meena, 2015; Fields and Friman, 2022; Hakim et al., 2021; Weller, 1988; Whipps, 2001). The composition of the rhizosphere microbial community and effects of rhizodeposition can be shaped by many factors including soil type, climate, disease, host species and genotype, and plant developmental age (Qu et al., 2020). In particular, rhizodeposition of sugars and other photosynthates can play an important role in driving the assembly of the rhizosphere microbial community under a wide range of conditions (Paterson et al., 2007). While bulk soil contains a relative dearth of microbially-available organic carbon, the rhizodeposition of carbon by a host plant creates a microenvironment with an elevated level of microbial activity (Starkey, 1938). Increases in carbon fixation play a key role in influencing the composition of the rhizosphere microbial community due to corresponding shifts in rhizodeposition (Pinton et al., 2007). However, as growth-defense tradeoffs require the reallocation of photosynthates to plant defense, the exclusion of foliar fungi is likely consequential for soil microbiome composition and functional capacities.

While host biomass and photosynthetic capacity are associated with the composition of the rhizosphere microbial community, it remains unclear, however, if increases in productivity resulting from foliar fungicide-associated alterations to plant growth-defense tradeoffs produce corresponding shifts in the composition of the rhizosphere microbial community. To answer this question, we harnessed experimental prairie plots where foliar fungicides or water controls have been applied continuously to determine the impact of foliar fungi on the productivity of perennial grasslands. After six years of continuous treatment, we sequenced the bacterial and fungal rhizosphere communities to determine how foliar fungal impacts on host productivity impact the composition of the rhizosphere microbial community. Here, we test the hypothesis that foliar fungicide-mediated increases in productivity are positively associated with the amplitude of compositional changes in the rhizosphere microbial community. Given the critical role the rhizosphere microbial community plays in plant health (Berendsen et al., 2012; Trivedi et al., 2020; Wallenstein, 2017), understanding the impacts of host infection and productivity on rhizosphere community assembly will shed light on potential mechanisms by which host plants influence rhizosphere microbial composition and functioning.

## Methods

### Field sites

Data used in this paper were collected from an experiment conducted in an experimental grassland at the University of Minnesota Cedar Creek Ecosystem Science Reserve (Latitude 45.4 N, Longitude 93.2 W; Cedar Creek), part of the U.S. Long-Term Ecological Research (LTER) Network. Cedar Creek has a temperate climate with a mean annual precipitation of 750 mm and mean annual temperature of 6°C. The soils are sandy and infertile compared to many other grasslands (Fay et al., 2015).

Each block of this experiment (9m x 9m) was planted in 1994 with seeds of 16 native prairie species: four C4 grasses, four C3 grasses, four legumes, and four forb species (for details of methods see Tilman et al., 2006). The field in which these blocks lie is permanently fenced to exclude vertebrate herbivores. Blocks are weeded annually to maintain plant species diversity and are burned annually in early spring to remove residual aboveground biomass. In 2008, two plots (1.5 m x 2 m) were established in each of six 16-species blocks, with half of the plots receiving biweekly water controls and half receiving applications of Quilt (Syngenta Crop Protection, Greensboro, NC, USA), a broad spectrum, systemic fungicide comprised of Azoxystrobin and Propiconazole (Borer et al., 2015). Note this experiment is a subset of a larger plant diversity and food web experiment described in detail by Borer et al. (2015) and Seabloom et al. (2017). Previous work from this larger experiment established that the aforementioned foliar fungicide regimen reduced disease severity approximately three-fold Borer et al. (2015). The block design of this experiment allows us to account for the well-documented impact of spatial location on rhizosphere microbial community composition and host growth (Christian et al., 2016; Finkel et al., 2011; Fitzpatrick et al., 2020; Klasek et al., 2023, 2021; Xing et al., 2023).

### Data collection

In 2014, we estimated aboveground biomass non-destructively in the fungicide and control plots of six blocks via reflected radiation using an MSR5 multispectral radiometer (Cropscan, Inc., Rochester, MN, USA). Measurements were taken for each plot approximately biweekly between May 28 – September 17, 2014. Measurements were taken 1m above the vegetation canopy as described by (Zaret et al., 2022). Season-long plot productivity was calculated as the area under the NDVI curve. Aboveground biomass was measured in early August 2014 by clipping, drying, and weighing a 0.1m x 1.0m strip from each plot (Seabloom et al., 2017; Zaret et al., 2024). Belowground plant biomass was estimated through the collection of three soil cores per plot (5cm diameter x 30cm depth), collected at peak biomass in August 2014, from random locations within each plot. Soil was washed from roots, and the roots were dried and weighed. NDVI, and belowground plant biomass were determined at the plot level. Seed production of individual plants was measured through the placement of nylon organza bags around the seed heads of ten randomly selected *Andropogon gerardii* and ten randomly selected *Lespedeza capitata* plants in late August, after fertilization but prior to seed maturation. In October, following natural senescence, we measured both the number and total dry weight of seeds produced per seed head and counted the total number of seed heads per plot.

To investigate the response of rhizosphere microbial communities to foliar fungicide application, soil cores (1cm diameter x 10cm depth) were collected from the base of three randomly selected *A. gerardii* and three randomly selected *L. capitata* (host plant) from each of the six foliar fungicide treated and six control plots in November of 2014. Triplicate rhizosphere soil samples were taken from the base of each individual host plant and bulked into a single composite sample for a total of six control and six foliar fungicide treated samples. A total of 72 composite rhizosphere samples were obtained (six blocks x two plots per block x two host species x three individuals per host species, with each sample composed of 3 bulked soil cores). Rhizosphere soil samples were transported in a cooler (4°C) to the lab, sieved on a 2mm screen, and stored at -20°C until processing. DNA was extracted using 0.25g of soil using the MoBio PowerSoil DNA extraction kit following the manufacturer’s instructions. DNA was quantified on a BioTek plate reader (Agilent Technologies, Santa Clara, CA, USA) and submitted to the University of Minnesota Genomics Center (UMGC) for amplification and sequencing. The fungal ITS1 region was amplified using the primers ITS1F:5’- CTTGGTCATTTAGAGGAAGTAA-3’ and ITS2:5’- GCTGCGTTCTTCATCGATGC-3’ using established protocols (Gohl et al., 2016; Smith and Peay, 2014). Amplicons were barcoded, pooled, and sequenced by Illumina Miseq 2x300 paired-end chemistry. The bacterial V5-V6 region of the bacterial 16S rRNA gene was amplified using the primers V5f:5’- RGGATTAGATACCC-3’ and V6r:5’- CGACRRCCATGCANCACCT-3’ using established protocols (Gohl et al., 2016). Amplicons were barcoded, pooled, and sequenced on an Illumina MiSeq machine using 2x250 paired-end chemistry.

Soil carbon, nitrogen, potassium, phosphorous, and pH were determined for each composite soil core collected from each plot. In brief, soil samples were dried overnight under sterilized cheesecloth. Total carbon and nitrogen were measured through combustion in a Costech CN elemental analyzer (Costech Analytical Technologies, Inc., Valencia, CA, USA). Soil pH was measured in a 1:2 (w/v) soil:water mixture using a Thermoscientific Orion Star A211 pH meter (Thermo Fisher Scientific, Waltham, MA, USA). Extractable potassium and phosphorous were measured at the University of Minnesota Research Analytical Laboratory, the methods are available at ral.cfans.umn.edu.

### Sequence processing

The fungal raw sequencing data were downloaded from the Data Repository for the University of Minnesota (DRUM, https://conservancy.umn.edu/), accession number 190418, utilizing the archives for 2014 data. Bacterial sequencing data has been deposited in the NCBI Short Read Archive under BioProject PRJNA1200703. Fungal and bacterial microbial community data were processed using QIIME2 (version 2023.2) as a wrapper for DADA2 unless otherwise noted (Bolyen et al., 2019; Callahan et al., 2016). In brief, demultiplexed reads were received with the forward and reverse primers already removed. Primer removal was confirmed using the *cutadapt trim-paired* command within QIIME2. Forward and reverse bacterial reads were truncated at 190 and 160 bp, corresponding with the degradation of quality scores observed at the 3’ end. Likewise, forward and reverse fungal reads were truncated at 230 and 170 bp respectively. Reads which were shorter than truncation values were discarded. Fungal and bacterial reads were quality filtered with expected errors less than 2.0 using DADA2 and clustered into OTUs at 97% similarity using *vsearch de novo* clustering within QIIME2. OTUs were used instead of ASVs as 97% similarity OTUs were the predominant method of addressing sequencing errors within the MiSeq chemistries used in this project. Taxonomic classification was assigned using *classify-sklearn*, a Naïve Bayes kmer classifier within the QIIME2 pipeline, trained on the UNITE 9.0 dynamic sequences (fungal) or the SILVA 138.1 (bacterial) database (Abarenkov et al., 2024; Quast et al., 2013). OTU tables and taxonomic classifications were exported to R for subsequent analyses.

### Sequence normalization

Rhizosphere samples were split across two Illumina MiSeq 2x250 (16S) and two 2x300 (ITS) runs for a total of four sequencing runs. Although samples were randomized to minimize sequencing batch effects, previous work with the data identified OTUs which were variably amplified between sequencing runs (Song et al., 2018). Three rhizosphere samples (110-1, 138-2A, and 182-2) were replicated in all sequencing runs, with each run containing three technical replicates of these rhizosphere samples. In total, the three rhizosphere samples were sequenced for 6 ITS and 6 rRNA technical replicates each. We identified variably amplified OTUs using a modified version of the approach described by Song et al. (2018). In brief, for each replicated rhizosphere sample (n=3) we converted total reads to relative abundance values. Within each replicated rhizosphere sample, we used a linear model to determine differential abundance between sequencing runs for each OTU. OTUs which were determined to be differentially abundant between sequencing runs (p<0.01) in any of the three replicated rhizosphere samples were excluded from the final OTU table. In total 29 bacterial and 28 fungal OTUs were excluded, comprising 4.0% and 3.1% of the total sequences, respectively. In addition, the *decontam* package (v1.16.0) (Davis et al., 2018) was used with two negative control samples for the identification of potential contaminating OTUs. The *decontam* package removed 4 bacterial and 24 fungal OTUs comprising 0.0125% and 0.878% of the sequences.

### Statistical analyses

All statistical analyses were conducted in R version 4.2.0 (R Core Team, 2022) using mixed effects models with p-values estimated using the package *lmerTest* (v3.1.3) (Kuznetsova et al., 2017) which provides p-values for random effects models using Satterthwaite’s degrees of freedom. Season-long aboveground plot productivity was calculated as the area under the NDVI curve. The impact of foliar fungicide application on plot belowground biomass, aboveground productivity, and seed measurements were compared using linear mixed-effect models, as implemented in *lmer*, with treatment as a fixed effect and plot as a random intercept to facilitate pairwise comparisons between paired plots. All data visualizations were produced using the package *ggplot2* (Wickham, 2016).

Data analysis was conducted in R using the packages *lmerTest*, *phyloseq* (v1.41.1), and *vegan* (v2.6.2) (Lin and Peddada, 2024; McMurdie and Holmes, 2013; Oksanen et al., 2022). Adequate sequencing depth was determined through visual inspection for saturation of rarefaction curves. The fungal and bacterial sequencing results obtained from four rhizosphere samples were removed due to insufficient sequencing depth, resulting in 68 total fungal and bacterial sequencing samples. Alpha diversity metrics (Chao1, observed OTUs, Shannon’s diversity index, and Pielou’s evenness) were calculated using samples rarefied to the depth of the sample with the smallest number of reads (7,615 fungal reads and 25,333 bacterial reads). We calculated Chao1, the number of observed OTUs, and Shannon’s diversity index using the *estimate_richness* function in the *phyloseq* package. Pielou’s evenness was calculated as a function of Shannon’s diversity and Chao1. α-diversity metrics were transformed and normality was confirmed through the Shapiro-Wilk test of normality and by visual inspections of the residuals. Shannon’s diversity and Pielou’s evenness were squared for normality, no transformation was necessary for Chao1 or observed OTUs. To test for differences in α-diversity metrics between paired treated and control plots we used a linear mixed effect model, implemented in the function *lmer*, on the transformed data, modeling treatment as a fixed effect and plot as a random intercept. The statistical significance of fixed effects was determined through an analysis of variance using the *anova* function.

To quantify variation in community composition, we scaled the number of reads assigned to each OTU to relative abundances within samples without rarefaction. Distance matrices were calculated with the *vegdist* function, as implemented in the package *vegan*, using Bray-Curtis and Jaccard dissimilarity metrics. The impact of foliar fungicide application and host species on rhizosphere microbial communities was determined through permutational multivariate analysis of variance (PERMANOVA) with the *adonis2* function using 25,000 permutations. Plot was used as a stratifying factor in PERMANOVA analyses due to the paired design of sampled plots. The amplitude of shifts in rhizosphere microbial communities, resulting from foliar fungicide application to each plot, was calculated with both Bray-Curtis and Jaccard dissimilarity as the mean iterative distance between all samples from paired foliar fungicide applied and control plots. Correlations between foliar-fungicide associated shifts in plot productivity and rhizosphere microbial communities were determined with linear models, as implemented in the function *lm*.

## Results

### Sequencing summary

#### Fungi

After quality filtering, 2.6 million reads were clustered into 1,900 OTUs at 97% identity. Of these, 127 OTUs were not assigned to the Kingdom Fungi and were removed from further analyses. Adequate sequencing depth of fungal samples was confirmed by visual inspections for saturation of rarefaction curves. The number of reads per sample ranged between 7,615 – 76,138 reads. The final OTU table was comprised of 1,773 OTUs and 2.5 million fungal reads.

#### Bacteria

We clustered 4.5 million bacterial reads into 17,085 OTUs at 97% sequence identity. Of these, 105 OTUs which were not assigned to Bacteria were removed. Adequate sequencing depth of the bacterial samples was confirmed by visual inspections for saturation of rarefaction curves. The number of reads per sample ranged between 25,333 – 239,163 reads. The final OTU table was comprised of 16,980 OTUs and 4.5 million bacterial reads.

### Impacts of foliar fungicide application on rhizosphere microbial diversity and composition

**Figure 1:**
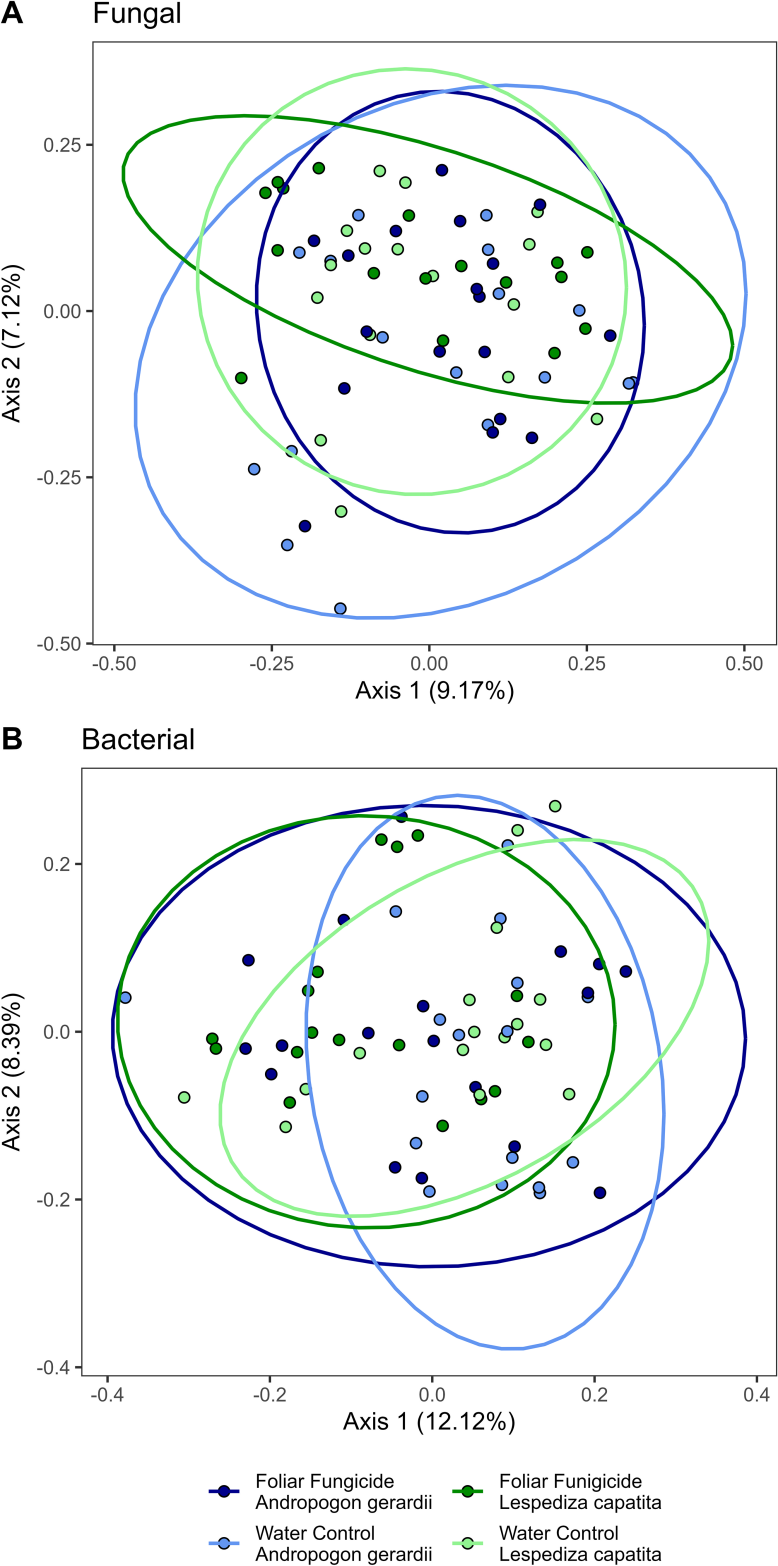
Principal coordinate analysis of **(A)** Fungal and **(B)** Bacterial populations using Bray-Curtis distances. Ellipses are drawn by treatment and host species.

Foliar fungicide applications shifted the species present in the community, as measured by Jaccard’s distance. However, the changes were primarily rare species, as we did not find differences when we used an abundance-weighted distance (Bray-Curtis) (**Table 1**). Rhizosphere fungal communities were also influenced by host species and the interaction of host species and foliar fungicide application. *Post-hoc* analysis revealed the rhizosphere fungal communities of *A. gerardii* and *L. capitata* differed only in plots which received foliar fungicide applications (**Figure 1**; **Supplementary Table S1**). In contrast to rhizosphere fungal communities, the composition of rhizosphere bacterial communities was altered by foliar fungicide application and host species, but not their interaction, regardless of dissimilarity metric (**Table 1**). Fungal and bacterial alpha diversity (richness, diversity, and evenness) were not impacted by foliar fungicide application (**Table 2**).

**Table 1:** Result of PERMANOVA investigating the impact of treatment, host species, and their interaction on the composition of the rhizosphere microbial community.

		Bray-Curtis		Jaccard	
		R^2^	p-value	R^2^	p-value
Fungal	Treatment	0.01801	0.06844	0.01748	**0.03748**
	Host Species	0.02147	**0.00768**	0.01929	**0.00604**
	Interaction	0.01998	**0.01788**	0.01849	**0.01236**
Bacterial	Treatment	0.02342	**0.00424**	0.02112	**0.00184**
	Host Species	0.01858	**0.02912**	0.01750	**0.02428**
	Interaction	0.01498	0.13323	0.01534	0.09436

PERMANOVAs were conducted using 25,000 permutations and paired subplots as a stratifying effect.

Bold p-values indicate significance.

**Table 2:** Analysis of the impact of (left) treatment and (middle) season-long subplot productivity on microbial diversity, as well as (right) the correlation of the percent change in season-long subplot productivity and change in diversity between paired treated and control subplots.

		Treatment		Correlation with subplot productivity		Change in paired subplot diversity *vs* changes in microbial diversity	
		F-value	p-value	F-value	p-value	F-value	p-value
Fungal	Observed OTUs	0.1733	0.6787	1.8720	0.1759	0.2352	0.6531
	Chao1	0.1810	0.6721	1.9494	0.1673	0.2987	0.6137
	Shannon’s Diversity	1.0934	0.2995	0.0300	0.8631	0.2070	0.6728
	Pielou’s Evenness	1.0280	0.3146	0.3735	0.5432	0.0506	0.8331
Bacterial	Observed OTUs	0.0744	0.7859	0.0112	0.9160	0.1757	0.6966
	Chao1	0.0601	0.8070	0.0198	0.8884	0.1981	0.6793
	Shannon’s Diversity	0.4681	0.4963	0.0345	0.8532	4.00E-04	0.9856
	Pielou’s Evenness	2.0040	0.1616	1.3184	0.2550	4.7436	0.0950

All analyses were conducted using a linear model. The analysis of treatment utilized paired treated and control subplots as a random effect. The analysis of subplot productivity utilized treatment as a random effect.

### Changes in aboveground plant productivity, but not aboveground biomass, are significantly correlated with shifts in rhizosphere microbial composition

**Figure 2:**
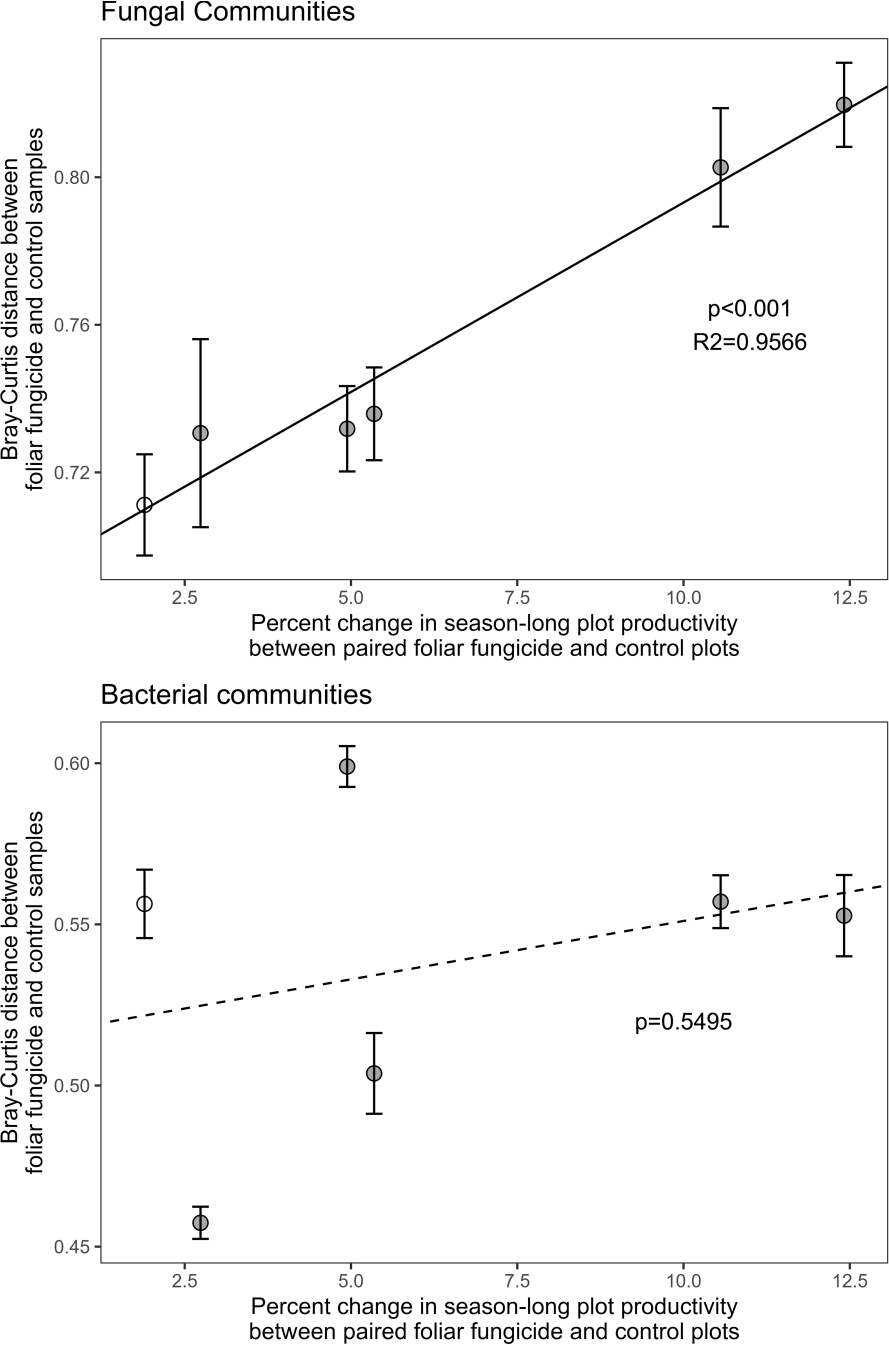
Correlation of the Bray-Curtis distance between paired foliar fungicide treated and control subplots with the absolute percent change in season-long productivity resulting from foliar fungicide application. Season-long productivity was measured as area under the NDVI curve from May 28 – September 17, 2014. Fungal communities are presented in the upper panel, bacterial communities in the lower panel. Solid dots indicate plots which increased in season-long productivity following foliar fungicide application, white dots decreased. Error bars represent standard error of microbial community change between paired plots.

The effect of foliar fungicide on season-long, aboveground plant productivity (area under the NDVI curve) was positively associated with the Bray-Curtis differences in rhizosphere fungal (p<0.001, R^2^ = 0.9566), but not bacterial communities (**Figure 2**) between paired treated and control plots. Rhizosphere bacterial communities exhibited a significantly smaller response to foliar fungicide application than fungal communities (bacterial Bray-Curtis dissimilarity between paired plots: 0.538 ± 0.0202 mean ± se, fungal 0.755 ± 0.0181; p<0.001). Changes in productivity associated with foliar fungicide application did not alter fungal alpha diversity, bacterial alpha diversity, or diversity shifts between paired plots (**Table 2**).

### Soil edaphic properties are associated with microbial communities and aboveground plant productivity but not the magnitude of fungicide mediated shifts

Over all plots, soil phosphorous and potassium were positively correlated with season-long aboveground plant productivity while pH was negatively correlated with productivity (**Supplementary Table S2**). However, fungicide treatments did not alter these relationships within blocks (**Supplementary Table S2**). While soil carbon, nitrogen, potassium, and pH were associated with significant differences in rhizosphere fungal or bacterial communities between paired plots (**Table 3**), the magnitude of soil chemical shifts between paired plots were not correlated with the amplitude of shifts in the rhizosphere microbial communities (**Table 3**).

**Table 3:** (Left) analyses of the impact of soil chemical properties on soil microbial composition as measured using a PERMANOVA with 25,000 permutations and stratifying by paired treated and control plots.

		Impact of soil chemical properties on the microbial community		Percent change in soil chemistry and microbial communities between paired subplots	
		R^2^	p-value	F-value	p-value
Fungal	Carbon	0.03918	**0.0041**	0.4356	0.5453
	Nitrogen	0.03540	**0.0128**	0.2813	0.6239
	Phosphorous	0.02030	0.4957	2.8758	0.1652
	Potassium	0.02343	**0.0160**	2.1190	0.2192
	pH	0.01793	0.8572	2.7898	0.1702
Bacteria	Carbon	0.02282	0.8886	5.7583	0.0744
	Nitrogen	0.02229	0.9118	6.6073	0.0620
	Phosphorous	0.02033	0.9700	0.3392	0.5916
	Potassium	0.03516	**1.00E-04**	0.0242	0.8839
	pH	0.04261	**0.0010**	0.5130	0.5134

(Right) Correlation of the percent change in soil chemical properties between paired subplots and the Bray-Curtis distance between the microbial communities of paired subplots. Comparisons were conducted using a linear model.

Bold p-values indicate significance.

### Foliar fungicide applications impact aboveground productivity and biomass but not root biomass or seed production.

**Figure 3:**
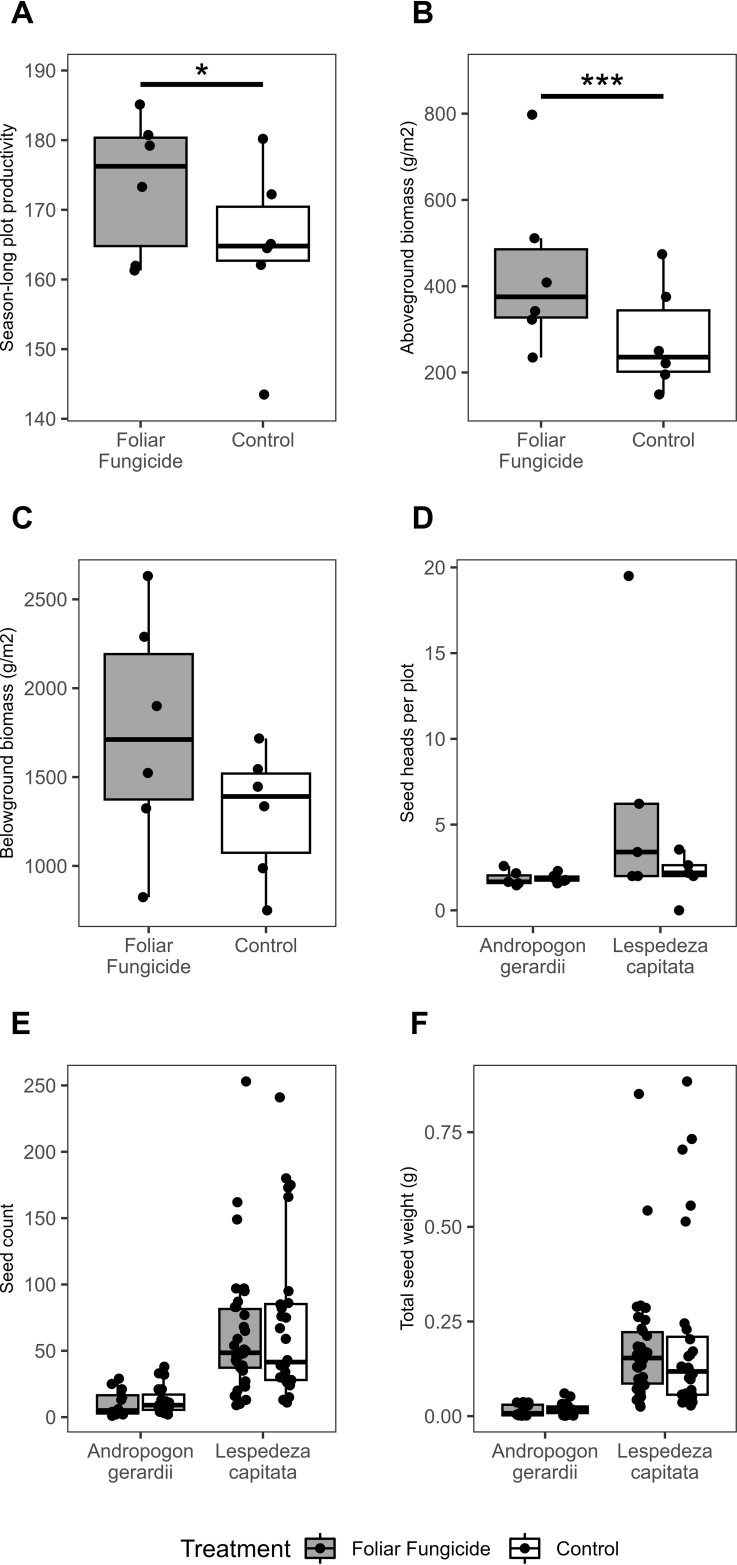
Impact of foliar fungicide application on **(A)** season-long productivity, **(B)** aboveground biomass, **(C)** belowground biomass, **(D)** seed heads per plot, **(E)** seed count, and **(F)** total seed weight. Season-long plot productivity was measured as area under the NDVI curve from May 28 – September 17, 2014. Asterisks within each panel represent significance: *p<0.05, ***p<0.001.

The application of foliar fungicide significantly increased season-long productivity and aboveground biomass of treated over paired control plots by an average of 5.7% and 59.1%, respectively (**Figures 3A, B**; **Supplementary Table S3**). In contrast, there were no significant differences in the peak-season belowground biomass, seed heads per plot, seed count per head, or seed weight per head between paired treated and control plots (**Figures 3C–F**; **Supplementary Table S3**). Season-long productivity, as measured by cumulative NDVI, was correlated with aboveground biomass (R^2^ = 0.6505), consistent with previous work (Zaret et al., 2022). However, changes in season-long productivity associated with foliar fungicide application were not significantly correlated with changes in aboveground biomass. This may reflect differences among plots in the relative timeline of biomass accumulation; a season long integrated metric (cumulative NDVI) *vs* a single end of season measurement (aboveground biomass).

## Discussion

The magnitude of change in aboveground plant productivity arising from foliar fungicide is associated with greater shifts in rhizosphere community composition. Foliar fungi can impose significant fitness costs upon host plants through infection, energy costs associated with plant host defense responses, and as non-pathogenic carbon sinks (Huot et al., 2014; Walters and Heil, 2007). Six years of fungal reduction via fungicides throughout each growing season increased season-long productivity but did not impact belowground biomass or seed production. Our results suggest the potential for increased productivity in response to reduced foliar fungal infection to be invested in root exudates, resulting in shifts in the rhizosphere microbial community.

Increases in NDVI are generally strongly correlated with increases in photosynthesis and increased production of sugars and other photosynthates (Gamon et al., 1995). However, after six years of treatments, we did not observe significant changes in belowground biomass or seed production in paired treated and control plots, suggesting that plants are not consistently increasing investment of photosynthates in reproduction or belowground biomass. Season-long productivity (cumulative NDVI) is correlated with host end-of-season aboveground biomass, consistent with previous work (Zaret et al., 2022). However, changes in season-long productivity following foliar fungicide application were not significantly correlated with changes in aboveground biomass in paired foliar fungicide treated *vs*. control plots, further suggesting that photosynthates were not solely invested in plant growth. Recent work has proposed rhizodeposition as a distinct classification of plant nutrient-use strategies (Guyonnet et al., 2018). Plants secrete sugars and other carbon compounds as root exudates in the form of metabolites, amino acids, mucilage, and cell lysates (Bais et al., 2006). This rhizodeposition can range from 5% – 44% of plant net carbon assimilation (Badri et al., 2013; Li et al., 2024; Paterson and Sim, 2000; Upadhyay et al., 2022) and can result in the selective enrichment of specific taxa within the rhizosphere microbial community (Barea et al., 2005). While we did not directly measure rhizodeposition in this study, root exudate profiles have been tied to both the composition and substrate preferences of rhizosphere-inhabiting microbes, putatively acting as a mechanism for host-mediated modifications of the rhizosphere community (Beattie, 2018; Lane et al., 2023a; Zhalnina et al., 2018). While alterations to the composition of the rhizosphere microbial community may result from influences other than rhizodeposition (e.g. soil texture and moisture, temperature, and spatial location), our comparisons of paired plots within the block design of this experiment allowed us to minimize the influence of influences not related to the biology of the host. Alterations to host rhizosphere investment and rhizosphere composition, as a response to modifications to the foliar fungal community, suggest potentially strong ties between above- and belowground plant health. Evidence from this study suggests that reducing the foliar pathogen burden does not necessarily result in altered belowground plant biomass or investment in reproduction, but rather, the substantial changes in the rhizosphere microbial community point to altered investment in root exudates.

The significant positive correlation of fungal but not bacterial communities with changes in season-long productivity may, in part, be explained by variation in how fungal and bacterial populations interact with host plant species. Huang et al. (2023) found that rhizosphere fungal communities are more sensitive to changes in soil carbon than are rhizosphere bacteria. The authors proposed that when faced with a dearth of labile carbon substrates the rhizosphere fungal community may become dominated by lignocellulose-degrading fungi. Increases in the deposition of comparably labile root exudates may, in turn, stimulate significant shifts in the rhizosphere fungal composition as the fungal community becomes dominated by fungi able to more rapidly utilize those substrates. As host species identity significantly impacts the rhizosphere microbial community through variation in root exudate composition, fungicide-associated increases in rhizodeposition may also explain why fungal communities were influenced by host species only in fungicide treated plots. In contrast to fungi, bacterial communities, which less commonly utilize lignocellulose organics, may undergo extended periods of dormancy in the absence of labile carbon substrates (Joergensen and Wichern, 2018). Because inactive or dead bacteria may still be detected by high-throughput sequencing, we cannot rule out the possibility that significant shifts in the composition or activity of bacterial communities in response to altered rhizodeposition occur in response to foliar fungicide treatment but were masked by abundant genetic material from dormant or dead bacteria (Carini et al., 2016).

Another potential explanation for the observed shifts in fungal rhizosphere communities could be possible direct impacts of fungicide application on the rhizosphere fungal microbiome, potentially through overspray or drip of foliar fungicide onto the soil despite protocols that explicitly avoided foliar runoff. The application of foliar fungicides, such as propiconazole, to the soil can result in significant changes to the microbial community (Sliti et al., 2024; Yen et al., 2009). However, most studies on the impact of foliar fungicides on the soil microbial community apply fungicides directly to the soil, potentially resulting in higher quantities of chemical residue, and potentially larger impacts, than would be observed through overspray or drip. In our study, the strong correlation we observed between changes in plot season-long productivity and the amplitude of shifts in the rhizosphere fungal community between paired plots implicates indirect effects drive the observed shifts in the rhizosphere fungal community. Further, the lack of clear convergence of fungicide-treated rhizosphere communities after repeated applications (**Supplementary Table S4**) suggests that shifts in the rhizosphere fungal community are driven by changes in plot productivity rather than direct fungicide impacts (Nettles et al., 2016; Santísima-Trinidad et al., 2018).

Rhizosphere microbial communities are important due to their potential to sustainably promote plant growth, aid in nutrient acquisition and soil carbon sequestration, and provide protection from pathogens and abiotic stress (Bano et al., 2021; Besset-Manzoni et al., 2018; Dessaux et al., 2016; Murali et al., 2021; Pii et al., 2015; Prashar et al., 2014; Solomon et al., 2024; Wang and Kuzyakov, 2024). However, researchers have broadly focused on foliar and rhizosphere microbial communities as distinct entities even on an individual host. Using a 6-year foliar fungicide study, we demonstrate that when treatments enhance aboveground plant productivity, this has substantial impacts on the rhizosphere microbiome. Future work should determine if 1) changes to the rhizosphere microbial community result from alterations to rhizodeposition, 2) if the observed modifications to the rhizosphere microbial community are functionally significant, and 3) if changes to the rhizosphere microbial community resulting from rhizodeposition are beneficial or detrimental to the host. Critically, however, this work suggests aboveground microbiomes significantly influence belowground microbial communities, potentially by altering plant investments in rhizodeposition and/or growth-defense tradeoffs.

## Data Availability

The datasets presented in this study can be found in online repositories. The names of the repository/repositories and accession number(s) can be found below: https://www.ncbi.nlm.nih.gov/, PRJNA1200703, https://conservancy.umn.edu/, 190418.

## References

[B1] Abarenkov K. Nilsson R. H. Larsson K.-H. Taylor A. F. S. May T. W. Frøslev T. G.. (2024). The UNITE database for molecular identification and taxonomic communication of fungi and other eukaryotes: sequences, taxa and classifications reconsidered. Nucleic Acids Res. 52, D791–D797. doi: 10.1093/nar/gkad1039 37953409 PMC10767974

[B2] Allen C. D. Macalady A. K. Chenchouni H. Bachelet D. McDowell N. Vennetier M.. (2010). A global overview of drought and heat-induced tree mortality reveals emerging climate change risks for forests. For. Ecol. Manage. 259, 660–684. doi: 10.1016/j.foreco.2009.09.001

[B3] Anderegg W. R. L. Schwalm C. Biondi F. Camarero J. J. Koch G. Litvak M.. (2015). Pervasive drought legacies in forest ecosystems and their implications for carbon cycle models. Science 349, 528–532. doi: 10.1126/science.aab1833 26228147

[B4] Badri D. V. Chaparro J. M. Zhang R. Shen Q. Vivanco J. M. (2013). Application of natural blends of phytochemicals derived from the root exudates of arabidopsis to the soil reveal that phenolic-related compounds predominantly modulate the soil microbiome*. J. Biol. Chem. 288, 4502–4512. doi: 10.1074/jbc.M112.433300 23293028 PMC3576057

[B5] Bai Y. Cotrufo M. F. (2022). Grassland soil carbon sequestration: Current understanding, challenges, and solutions. Science 377, 603–608. doi: 10.1126/science.abo2380 35926033

[B6] Bais H. P. Weir T. L. Perry L. G. Gilroy S. Vivanco J. M. (2006). The role of root exudates in rhizosphere interactions with plants and other organisms. Annu. Rev. Plant Biol. 57, 233–266. doi: 10.1146/annurev.arplant.57.032905.105159 16669762

[B7] Bano S. Wu X. Zhang X. (2021). Towards sustainable agriculture: rhizosphere microbiome engineering. Appl. Microbiol. Biotechnol. 105, 7141–7160. doi: 10.1007/s00253-021-11555-w 34508284

[B8] Barea J.-M. Pozo M. J. Azcón R. Azcón-Aguilar C. (2005). Microbial co-operation in the rhizosphere. J. Exp. Bot. 56, 1761–1778. doi: 10.1093/jxb/eri197 15911555

[B9] Bazzaz F. A. Grace J. (1997). Plant Resource Allocation. San Diego: Academic Press.

[B10] Beattie G. A. (2018). Metabolic coupling on roots. Nat. Microbiol. 3, 396–397. doi: 10.1038/s41564-018-0139-1 29588539

[B11] Berendsen R. L. Pieterse C. M. J. Bakker P. A. H. M. (2012). The rhizosphere microbiome and plant health. Trends Plant Sci. 17, 478–486. doi: 10.1016/j.tplants.2012.04.001 22564542

[B12] Besset-Manzoni Y. Rieusset L. Joly P. Comte G. Prigent-Combaret C. (2018). Exploiting rhizosphere microbial cooperation for developing sustainable agriculture strategies. Environ. Sci. pollut. Res. 25, 29953–29970. doi: 10.1007/s11356-017-1152-2 29313197

[B13] Bolyen E. Rideout J. R. Dillon M. R. Bokulich N. A. Abnet C. C. Al-Ghalith G. A.. (2019). Reproducible, interactive, scalable and extensible microbiome data science using QIIME 2. Nat. Biotechnol. 37, 852–857. doi: 10.1038/s41587-019-0209-9 31341288 PMC7015180

[B14] Borer E. T. Lind E. M. Ogdahl E. J. Seabloom E. W. Tilman D. Montgomery R. A.. (2015). Food-web composition and plant diversity control foliar nutrient content and stoichiometry ed. Amy Zanne. J. Ecol. 103, 1432–1441. doi: 10.1111/1365-2745.12461

[B15] Callahan B. J. McMurdie P. J. Rosen M. J. Han A. W. Johnson A. J. A. Holmes S. P. (2016). DADA2: High-resolution sample inference from Illumina amplicon data. Nat. Methods 13, 581–583. doi: 10.1038/nmeth.3869 27214047 PMC4927377

[B16] Carini P. Marsden P. J. Leff J. W. Morgan E. E. Strickland M. S. Fierer N. (2016). Relic DNA is abundant in soil and obscures estimates of soil microbial diversity. Nat. Microbiol. 2, 16242. doi: 10.1038/nmicrobiol.2016.242 27991881

[B17] Carretero R. Bancal M. O. Miralles D. J. (2011). Effect of leaf rust (Puccinia triticina) on photosynthesis and related processes of leaves in wheat crops grown at two contrasting sites and with different nitrogen levels. Eur. J. Agron. 35, 237–246. doi: 10.1016/j.eja.2011.06.007

[B18] Cesarino I. (2019). Structural features and regulation of lignin deposited upon biotic and abiotic stresses. Curr. Opin. Biotechnol. 56, 209–214. doi: 10.1016/j.copbio.2018.12.012 30684783

[B19] Christian N. Sullivan C. Visser N. D. Clay K. (2016). Plant host and geographic location drive endophyte community composition in the face of perturbation. Microb. Ecol. 72, 621–632. doi: 10.1007/s00248-016-0804-y 27341838

[B20] Dass P. Houlton B. Z. Wang Y. Warlind D. (2018). Grasslands may be more reliable carbon sinks than forests in California. Environ. Res. Lett. 13, 074027. doi: 10.1088/1748-9326/aacb39

[B21] Davis N. M. Proctor D. M. Holmes S. P. Relman D. A. Callahan B. J. (2018). Simple statistical identification and removal of contaminant sequences in marker-gene and metagenomics data. Microbiome 6, 226. doi: 10.1186/s40168-018-0605-2 30558668 PMC6298009

[B22] Dessaux Y. Grandclément C. Faure D. (2016). Engineering the rhizosphere. Trends Plant Sci. 21, 266–278. doi: 10.1016/j.tplants.2016.01.002 26818718

[B23] Dotaniya M. L. Meena V. D. (2015). Rhizosphere effect on nutrient availability in soil and its uptake by plants: A review. Proc. Natl. Acad. Sci. India Sect. B Biol. Sci. 85, 1–12. doi: 10.1007/s40011-013-0297-0

[B24] Duffy J. E. Cardinale B. J. France K. E. McIntyre P. B. Thébault E. Loreau M. (2007). The functional role of biodiversity in ecosystems: incorporating trophic complexity. Ecol. Lett. 10, 522–538. doi: 10.1111/j.1461-0248.2007.01037.x 17498151

[B25] Fay P. A. Prober S. M. Harpole W. S. Knops J. M. H. Bakker J. D. Borer E. T.. (2015). Grassland productivity limited by multiple nutrients. Nat. Plants 1, 15080. doi: 10.1038/nplants.2015.80 27250253

[B26] Fields B. Friman V.-P. (2022). Microbial eco-evolutionary dynamics in the plant rhizosphere. Curr. Opin. Microbiol. 68, 102153. doi: 10.1016/j.mib.2022.102153 35504054

[B27] Finkel O. M. Burch A. Y. Lindow S. E. Post A. F. Belkin S. (2011). Geographical location determines the population structure in phyllosphere microbial communities of a salt-excreting desert tree. Appl. Environ. Microbiol. 77, 7647–7655. doi: 10.1128/AEM.05565-11 21926212 PMC3209174

[B28] Fitzpatrick C. R. Salas-González I. Conway J. M. Finkel O. M. Gilbert S. Russ D.. (2020). The plant microbiome: from ecology to reductionism and beyond. Annu. Rev. Microbiol. 74, 81–100. doi: 10.1146/annurev-micro-022620-014327 32530732

[B29] Gamon J. A. Field C. B. Goulden M. L. Griffin K. L. Hartley A. E. Joel G.. (1995). Relationships between NDVI, canopy structure, and photosynthesis in three Californian vegetation types. Ecol. Appl. 5, 28–41. doi: 10.2307/1942049

[B30] Gohl D. M. Vangay P. Garbe J. MacLean A. Hauge A. Becker A.. (2016). Systematic improvement of amplicon marker gene methods for increased accuracy in microbiome studies. Nat. Biotechnol. 34, 942–949. doi: 10.1038/nbt.3601 27454739

[B31] Guyonnet J. P. Cantarel A. A. M. Simon L. Haichar F. el Z. (2018). Root exudation rate as functional trait involved in plant nutrient-use strategy classification. Ecol. Evol. 8, 8573–8581. doi: 10.1002/ece3.4383 30250724 PMC6144958

[B32] Hakim S. Naqqash T. Nawaz M. S. Laraib I. Siddique M. J. Zia R.. (2021). Rhizosphere engineering with plant growth-promoting microorganisms for agriculture and ecological sustainability. Front. Sustain. Food Syst. 5. doi: 10.3389/fsufs.2021.617157

[B33] Huang Z. He X. Zhang C. Zhang M. Wang J. Hou Y.. (2023). Microbial communities and functions changed in rhizosphere soil of Pinus massoniana provenances with different carbon storage. Front. Microbiol. 14. doi: 10.3389/fmicb.2023.1264670 PMC10655096 38029152

[B34] Hungate B. A. Holland E. A. Jackson R. B. Chapin F. S. Mooney H. A. Field C. B. (1997). The fate of carbon in grasslands under carbon dioxide enrichment. Nature 388, 576–579. doi: 10.1038/41550

[B35] Huot B. Yao J. Montgomery B. L. He S. Y. (2014). Growth–defense tradeoffs in plants: A balancing act to optimize fitness. Mol. Plant 7, 1267–1287. doi: 10.1093/mp/ssu049 24777989 PMC4168297

[B36] Joergensen R. G. Wichern F. (2018). Alive and kicking: Why dormant soil microorganisms matter. Soil Biol. Biochem. 116, 419–430. doi: 10.1016/j.soilbio.2017.10.022

[B37] Jones D. L. Hodge A. Kuzyakov Y. (2004). Plant and mycorrhizal regulation of rhizodeposition. New Phytol. 163, 459–480. doi: 10.1111/j.1469-8137.2004.01130.x 33873745

[B38] Klasek S. A. Brock M. T. Morrison H. G. Weinig C. Maignien L. (2021). Soil microsite outweighs cultivar genotype contribution to brassica rhizobacterial community structure. Front. Microbiol. 12. doi: 10.3389/fmicb.2021.645784 PMC8058099 33897658

[B39] Klasek S. Crants J. Abbas T. Ashley K. A. Bolton M. Celovsky M.. (2023). Potato soil core microbiomes are regionally variable across the continental US. Phytobiom. J. PBIOMES-07-23-0060-R. 8 (2), 168–178. doi: 10.1094/PBIOMES-07-23-0060-R

[B40] Kumudini S. Prior E. Omielan J. Tollenaar M. (2008). Impact of *phakopsora pachyrhizi* infection on soybean leaf photosynthesis and radiation absorption. Crop Sci. 48, 2343–2350. doi: 10.2135/cropsci2008.05.0258

[B41] Kuznetsova A. Brockhoff P. B. Christensen R. H. B. (2017). lmerTest package: tests in linear mixed effects models. J. Stat. Soft. 82, 1–26. doi: 10.18637/jss.v082.i13

[B42] Lane B. R. Anderson H. M. Dicko A. H. Fulcher M. R. Kinkel L. L. (2023a). Temporal variability in nutrient use among Streptomyces suggests dynamic niche partitioning. Environ. Microbiol. 25, 3527–3535. doi: 10.1111/1462-2920.16498 37669222

[B43] Lane B. R. Kendig A. E. Wojan C. M. Adhikari A. Jusino M. A. Kortessis N.. (2023b). Fungicide-mediated shifts in the foliar fungal community of an invasive grass. Phytobiom. J. 7, 198–207. doi: 10.1094/PBIOMES-03-22-0018-R

[B44] Li H. Chang L. Liu H. Li Y. (2024). Diverse factors influence the amounts of carbon input to soils via rhizodeposition in plants: A review. Sci. Total Environ. 948, 174858. doi: 10.1016/j.scitotenv.2024.174858 39034011

[B45] Lin H. Peddada S. D. (2024). Multigroup analysis of compositions of microbiomes with covariate adjustments and repeated measures. Nat. Methods 21, 83–91. doi: 10.1038/s41592-023-02092-7 38158428 PMC10776411

[B46] McDowell N. G. Allen C. D. (2015). Darcy’s law predicts widespread forest mortality under climate warming. Nat. Clim. Change 5, 669–672. doi: 10.1038/nclimate2641

[B47] McMurdie P. J. Holmes S. (2013). phyloseq: an R package for reproducible interactive analysis and graphics of microbiome census data ed. Michael watson. PloS One 8, e61217. doi: 10.1371/journal.pone.0061217 23630581 PMC3632530

[B48] Murali M. Naziya B. Ansari M. A. Alomary M. N. AlYahya S. Almatroudi A.. (2021). Bioprospecting of rhizosphere-resident fungi: their role and importance in sustainable agriculture. JoF 7, 314. doi: 10.3390/jof7040314 33919629 PMC8072672

[B49] Nettles R. Watkins J. Ricks K. Boyer M. Licht M. Atwood L. W.. (2016). Influence of pesticide seed treatments on rhizosphere fungal and bacterial communities and leaf fungal endophyte communities in maize and soybean. Appl. Soil Ecol. 102, 61–69. doi: 10.1016/j.apsoil.2016.02.008

[B50] O’Brien J. A. Daudi A. Butt V. S. Paul Bolwell G. (2012). Reactive oxygen species and their role in plant defence and cell wall metabolism. Planta 236, 765–779. doi: 10.1007/s00425-012-1696-9 22767200

[B51] Oksanen J. Simpson G. L. Blanchet F. G. Kindt R. Legendre P. Minchin P. R.. (2022). vegan: Community Ecology Package version 2.6-2 April 2022. The Comprehensive R Archive Network. Available online at: http://cran.r-project.org.

[B52] Paterson E. Gebbing T. Abel C. Sim A. Telfer G. (2007). Rhizodeposition shapes rhizosphere microbial community structure in organic soil. New Phytol. 173, 600–610. doi: 10.1111/j.1469-8137.2006.01931.x 17244055

[B53] Paterson E. Sim A. (2000). Effect of nitrogen supply and defoliation on loss of organic compounds from roots of Festuca rubra. J. Exp. Bot. 51, 1449–1457. doi: 10.1093/jexbot/51.349.1449 10944159

[B54] Pieterse C. M. J. Zamioudis C. Berendsen R. L. Weller D. M. Van Wees S. C. M. Bakker P. A. H. M. (2014). Induced systemic resistance by beneficial microbes. Annu. Rev. Phytopathol. 52, 347–375. doi: 10.1146/annurev-phyto-082712-102340 24906124

[B55] Pii Y. Mimmo T. Tomasi N. Terzano R. Cesco S. Crecchio C. (2015). Microbial interactions in the rhizosphere: beneficial influences of plant growth-promoting rhizobacteria on nutrient acquisition process. A review. Biol. Fertil. Soils 51, 403–415. doi: 10.1007/s00374-015-0996-1

[B56] Pinton R. Varanini Z. Nannipieri P. (Eds.) (2007). The Rhizosphere: Biochemistry and Organic Substances at the Soil-Plant Interface. 2nd ed. (Boca Raton: CRC Press). doi: 10.1201/9781420005585

[B57] Prashar P. Kapoor N. Sachdeva S. (2014). Rhizosphere: its structure, bacterial diversity and significance. Rev. Environ. Sci. Biotechnol. 13, 63–77. doi: 10.1007/s11157-013-9317-z

[B58] Qu Q. Zhang Z. Peijnenburg W. J. G. M. Liu W. Lu T. Hu B.. (2020). Rhizosphere microbiome assembly and its impact on plant growth. J. Agric. Food Chem. 68, 5024–5038. doi: 10.1021/acs.jafc.0c00073 32255613

[B59] Quast C. Pruesse E. Yilmaz P. Gerken J. Schweer T. Yarza P.. (2013). The SILVA ribosomal RNA gene database project: improved data processing and web-based tools. Nucleic Acids Res. 41, D590–D596. doi: 10.1093/nar/gks1219 23193283 PMC3531112

[B60] R Core Team. (2022). R: A language and environment for statistical computing. Vienna, Austria: R Foundation for Statistical Computing. Available online at: https://www.R-project.org/.

[B61] Rodriguez R. J. White J. F. Jr. Arnold A. E. Redman R. S. (2009). Fungal endophytes: diversity and functional roles. New Phytol. 182, 314–330. doi: 10.1111/j.1469-8137.2009.02773.x 19236579

[B62] Ruuskanen S. Fuchs B. Nissinen R. Puigbò P. Rainio M. Saikkonen K.. (2023). Ecosystem consequences of herbicides: the role of microbiome. Trends Ecol. Evol. 38, 35–43. doi: 10.1016/j.tree.2022.09.009 36243622

[B63] Santísima-Trinidad A. B. L. del-Mar-Montiel-Rozas M. Diéz-Rojo M.Á. Pascual J. A. Ros M. (2018). Impact of foliar fungicides on target and non-target soil microbial communities in cucumber crops. Ecotoxicol. Environ. Saf. 166, 78–85. doi: 10.1016/j.ecoenv.2018.09.074 30248564

[B64] Seabloom E. W. Kinkel L. Borer E. T. Hautier Y. Montgomery R. A. Tilman D. (2017). Food webs obscure the strength of plant diversity effects on primary productivity ed. Brenda Casper. Ecol. Lett. 20, 505–512. doi: 10.1111/ele.12754 28295970

[B65] Sliti A. Singh V. Ibal J. C. Jeong M. Shin J.-H. (2024). Impact of propiconazole fungicide on soil microbiome (bacterial and fungal) diversity, functional profile, and associated dehydrogenase activity. Environ. Sci. pollut. Res. 31, 8240–8253. doi: 10.1007/s11356-023-31643-w 38175519

[B66] Smith D. P. Peay K. G. (2014). Sequence depth, not PCR replication, improves ecological inference from next generation DNA sequencing. PloS One 9, e90234. doi: 10.1371/journal.pone.0090234 24587293 PMC3938664

[B67] Solomon W. Janda T. Molnár Z. (2024). Unveiling the significance of rhizosphere: Implications for plant growth, stress response, and sustainable agriculture. Plant Physiol. Biochem. 206, 108290. doi: 10.1016/j.plaphy.2023.108290 38150841

[B68] Song Z. Schlatter D. Gohl D. M. Kinkel L. L. (2018). Run-to-run sequencing variation can introduce taxon-specific bias in the evaluation of fungal microbiomes. Phytobiom. J. 2, 165–170. doi: 10.1094/PBIOMES-09-17-0041-R

[B69] Starkey R. L. (1938). Some influences of the development of higher plants upon the microorganisms in the soil: VI. Microscopic examination of the rhizosphere. Soil Sci. 45, 207. doi: 10.1097/00010694-193803000-00005

[B70] Tian T. Reverdy A. She Q. Sun B. Chai Y. (2020). The role of rhizodeposits in shaping rhizomicrobiome. Environ. Microbiol. Rep. 12, 160–172. doi: 10.1111/1758-2229.12816 31858707

[B71] Tilman D. Reich P. B. Knops J. M. H. (2006). Biodiversity and ecosystem stability in a decade-long grassland experiment. Nature 441, 629–632. doi: 10.1038/nature04742 16738658

[B72] Trivedi P. Leach J. E. Tringe S. G. Sa T. Singh B. K. (2020). Plant–microbiome interactions: from community assembly to plant health. Nat. Rev. Microbiol. 18, 607–621. doi: 10.1038/s41579-020-0412-1 32788714

[B73] Upadhyay S. K. Srivastava A. K. Rajput V. D. Chauhan P. K. Bhojiya A. A. Jain D.. (2022). Root exudates: mechanistic insight of plant growth promoting rhizobacteria for sustainable crop production. Front. Microbiol. 13. doi: 10.3389/fmicb.2022.916488 PMC9329127 35910633

[B74] Van Hulten M. Pelser M. Van Loon L. C. Pieterse C. M. J. Ton J. (2006). Costs and benefits of priming for defense in *Arabidopsis* . Proc. Natl. Acad. Sci. U.S.A. 103, 5602–5607. doi: 10.1073/pnas.0510213103 16565218 PMC1459400

[B75] Vicente-Serrano S. M. Gouveia C. Camarero J. J. Beguería S. Trigo R. López-Moreno J. I.. (2013). Response of vegetation to drought time-scales across global land biomes. Proc. Natl. Acad. Sci. U.S.A. 110, 52–57. doi: 10.1073/pnas.1207068110 23248309 PMC3538253

[B76] Vives-Peris V. de Ollas C. Gómez-Cadenas A. Pérez-Clemente R. M. (2020). Root exudates: from plant to rhizosphere and beyond. Plant Cell Rep. 39, 3–17. doi: 10.1007/s00299-019-02447-5 31346716

[B77] Wallenstein M. D. (2017). Managing and manipulating the rhizosphere microbiome for plant health: A systems approach. Rhizosphere 3, 230–232. doi: 10.1016/j.rhisph.2017.04.004

[B78] Walters D. Heil M. (2007). Costs and trade-offs associated with induced resistance. Physiol. Mol. Plant Pathol. 71, 3–17. doi: 10.1016/j.pmpp.2007.09.008

[B79] Walters D. R. Paterson L. Walsh D. J. Havis N. D. (2008). Priming for plant defense in barley provides benefits only under high disease pressure. Physiol. Mol. Plant Pathol. 73, 95–100. doi: 10.1016/j.pmpp.2009.03.002

[B80] Wang C. Kuzyakov Y. (2024). Rhizosphere engineering for soil carbon sequestration. Trends Plant Sci. 29, 447–468. doi: 10.1016/j.tplants.2023.09.015 37867041

[B81] Weller D. M. (1988). Biological control of soilborne plant pathogens in the rhizosphere with bacteria. Annu. Rev. Phytopathol. 26, 379–407. doi: 10.1146/annurev.py.26.090188.002115

[B82] Whipps J. M. (2001). Microbial interactions and biocontrol in the rhizosphere. J. Exp. Bot. 52, 487–511. doi: 10.1093/jexbot/52.suppl_1.487 11326055

[B83] Wickham H. (2016). ggplot2: elegant graphics for data analysis. 2nd ed. (Switzerland: Springer). doi: 10.1007/978-3-319-24277-4

[B84] Xing Y. Bian C. Xue H. Song Y. Men W. Hou W.. (2023). The effect of plant compartment and geographical location on shaping microbiome of Pulsatilla chinensis. Appl. Microbiol. Biotechnol. 107, 5555–5567. doi: 10.1007/s00253-023-12641-x 37436481

[B85] Yen J.-H. Chang J.-S. Huang P.-J. Wang Y.-S. (2009). Effects of fungicides triadimefon and propiconazole on soil bacterial communities. J. Environ. Sci. Health Part B 44, 681–689. doi: 10.1080/03601230903163715 20183078

[B86] Yu Z. Lu T. Qian H. (2023). Pesticide interference and additional effects on plant microbiomes. Sci. Total Environ. 888, 164149. doi: 10.1016/j.scitotenv.2023.164149 37196943

[B87] Zaret M. Kinkel L. Borer E. T. Seabloom E. W. (2024). Plant growth–defense trade-offs are general across interactions with fungal, insect, and mammalian consumers. Ecology 105, e4290. doi: 10.1002/ecy.4290 38570923

[B88] Zaret M. M. Kuhs M. A. Anderson J. C. Seabloom E. W. Borer E. T. Kinkel L. L. (2022). Seasonal shifts from plant diversity to consumer control of grassland productivity ed. Vojtech Novotny. Ecol. Lett. 25, 1215–1224. doi: 10.1111/ele.13993 35229976 PMC9544143

[B89] Zhalnina K. Louie K. B. Hao Z. Mansoori N. da Rocha U. N. Shi S.. (2018). Dynamic root exudate chemistry and microbial substrate preferences drive patterns in rhizosphere microbial community assembly. Nat. Microbiol. 3, 470–480. doi: 10.1038/s41564-018-0129-3 29556109

