# Supplementary material for: Foliar fungi-imposed costs to plant productivity moderate shifts in composition of the rhizosphere microbiome
Source: Front Plant Sci. 2025 Mar 5;16:1558191. doi: 10.3389/fpls.2025.1558191 (PMC11921152; doi:10.3389/fpls.2025.1558191)
Supplement: Supplementary file 1 [file Table1.docx]

Table S1. Result of PERMANOVA investigating the impact of host species on the composition of the rhizosphere fungal community. PERMANOVAs were conducted using 25,000 permutations.

|  |  | Bray-Curtis | |  | Jaccard | |
| --- | --- | --- | --- | --- | --- | --- |
|  |  | R^2^ | p-value |  | R^2^ | p-value |
| Foliar Fungicide Plots | Species | 0.05112 | **4.00E-05** |  | 0.04422 | **8.00E-05** |
| Control Plots | Species | 0.03312 | 0.1871 |  | 0.03249 | 0.1837 |

Table S2. (Left) Correlation of soil chemical properties and season-long subplot productivity. (Right) Relationship of the percent change in season-long productivity between paired plots and the percent change in soil chemical properties between paired plots. All comparisons were conducted using a linear model.

|  | By individual plots | |  | By paired plots | |
| --- | --- | --- | --- | --- | --- |
|  | F-value | p-value |  | F-value | p-value |
| Carbon | 0.0751 | 0.785 |  | 0.4356 | 0.5453 |
| Nitogen | 0.1389 | 0.7106 |  | 0.2813 | 0.6239 |
| Phosphorous | 41.532 | **1.56E-08** |  | 2.8758 | 0.1652 |
| Potassium | 11.934 | **0.000968** |  | 2.119 | 0.2192 |
| pH | 5.6498 | **0.02037** |  | 2.7898 | 0.1702 |

Table S3. Impact of foliar fungicide application on season-long productivity, above- and belowground biomass, seed count, and total seed weight. Analyses were conducted using a mixed effect linear model with treatment as a fixed effect and paired plots as a random effect.

|  |  | F-value | p-value |
| --- | --- | --- | --- |
| Season-long productivity | | 7.9152 | **0.0374** |
| Aboveground biomass | | 16.845 | **0.0093** |
| Belowground biomass | | 2.1601 | 0.1724 |
| Seed count | *A. gerardii* | 1.4737 | 0.2355 |
|  | *L. capitata* | 0.4721 | 0.4947 |
| Total seed weight | *A. gerardii* | 0.1637 | 0.2118 |
|  | *L. capitata* | 0.2950 | 0.5890 |
| Total seed heads | *A. gerardii* | 0.0251 | 0.8803 |
|  | *L. capitata* | 1.8337 | 0.2127 |

Table S4. Analysis of the impact of plot on microbial community composition using Bray-Curtis and Jaccard distances. Foliar fungicide and control plot analyses were conducted separately. PERMANOVAs were conducted using 25,000 permutations.

|  | Bray-Curtis | |  | Jaccard | |
| --- | --- | --- | --- | --- | --- |
|  | R^2^ | p-value |  | R^2^ | p-value |
| Foliar Fungicide | 0.05070 | **0.00424** |  | 0.04212 | **0.00556** |
| Control | 0.05029 | **0.01156** |  | 0.04415 | **0.00780** |
